# Impact of Fennel Essential Oil as an Antibiotic Alternative in Rabbit Diet on Antioxidant Enzymes Levels, Growth Performance, and Meat Quality

**DOI:** 10.3390/antiox10111797

**Published:** 2021-11-10

**Authors:** Tharwat Imbabi, Islam Sabeq, Ali Osman, Kamal Mahmoud, Shimaa A. Amer, Aziza M. Hassan, Nikolay Kostomakhin, Walid Habashy, Ahmed A. Easa

**Affiliations:** 1 Department of Animal Production, Faculty of Agriculture, Benha Univerisity, Benha 13736, Egypt; 2 Department of Food Hygiene, Faculty of Veterinary Medicine, Benha University, Benha 13736, Egypt; Islam.sabek@fvtm.bu.edu.eg; 3 Biochemistry Department, Faculty of Agriculture, Zagazig University, Zagazig 44511, Egypt; 4 Biochemistry Department, Faculty of Agriculture, Menoufia University, Shibin El-Kom 32511, Egypt; Kemam_mahmoud@agr.menofia.edu.eg; 5 Department of Nutrition & Clinical Nutrition, Faculty of Veterinary Medicine, Zagazig University, Zagazig 44511, Egypt; shimaa.amer@zu.edu.eg; 6 Department of Biotechnology, College of Science, Taif University, P.O. Box 11099, Taif 21944, Saudi Arabia; a.hasn@tu.edu.sa; 7 Department of Dairy and Beef Cattle Breeding, Russian State Agrarian University-Moscow Agriculture Academy, 115432 Moscow, Russia; kostomakhin@rgau-msha.ru; 8 Faculty of Agriculture, Animal and Poultry Production, Damanhour University, Damanhour 22511, Egypt; walid.habashi@agr.dmu.edu.eg (W.H.); ahmed.allam@agr.dmu.edu.eg (A.A.E.)

**Keywords:** weaned rabbits, fennel oil, apoptosis, gene expression, meat quality

## Abstract

In the current study, fennel essential oil was used as an antibiotic alternative compared to gentamycin for enhancing the expression of apoptosis genes and antioxidant enzymes in weaned rabbits as well as meat quality and growth performance. The gene expression of the cell lymphoma 2 (BAX and BCL2), caspase3 (CASP3), and glutathione peroxidase (GPX1) were estimated in the liver tissue using qRT-PCR. A total of 45 Moshtohor weaned male rabbits aged four weeks were randomly allocated to control, T1, and T2 treatment groups; each consisted of 15 weaned male rabbits with five replicates. Rabbits in the T1 and T2 groups were orally supplied with 1 mL fennel oil and 1 mL gentamycin, respectively. Weaned rabbits under different treatments showed increased body weight (BW) at 8 and 12 weeks of age and average daily gain (ADG) at 4–8 and 4–12 weeks of age compared to the control group. Compared to the controls, the weaned rabbits supplemented with fennel oil and gentamycin had lower total cholesterol, triglyceride, and MDA. In addition, villus length, mRNA of BAX, BCL2, Casp3, and GPX were increased in the different treatments compared to the control. Furthermore, the meat of these rabbits was less tender, had a lower aerobic plate count (APC), pH, and was brighter and redder in color than the control. Under the conditions of the present study, the supplementation of weaned Moshtohor rabbits with fennel oil as a natural alternative for gentamycin enhanced feed conversion and daily gain through enhancing villus length and mucus thickness. Additionally, fennel essential oil reduces oxidative stress by increasing the antioxidant enzymes.

## 1. Introduction

Antibiotics remain the most effective tool for treating animal diseases and promoting growth, but safe alternatives, such as feed additives, must be provided to reduce their use in farm animals and achieve better livestock production results. In rabbit farming, weaning rabbits are affected by many digestive diseases [1], which increase mortality rate [2], and, in turn, causes the antibiotics to be used more intensively. Gentamycin is an aminoglycoside derived from *Micomonospora puerperal* [3]. Khaki et al. [4] reported that gentamycin has a strong effect against gram-negative bacteria. Recently, many countries have already banned antibiotics for their side effects on both animals and humans. Thus, innovative feed supplements which can improve feed utilization and health status are needed; in addition to their antipathogenic effect, they act as antioxidants and immune response stimulants such as probiotics, organic acids, symbiotic, enzymes, medical plants, basic proteins, modified proteins, natural pigments, and their extracts [5,6,7,8,9,10,11,12,13,14,15]. Essential oil is a concentrated hydrophobic liquid made up of volatile chemical components derived from plants. Essential oils (Eos) as one group of phytogenic feed additives [16,17] play a vital role in promoting the digestion phase through enhancing the secretion of digestive fluids, enzyme stimulation, and reducing the effects of pathogenic bacteria [18,19]. In addition, Eos have an antibacterial effect due to their ability to enter the bacteria through the cell membrane, leading to a change in cell structure and functional properties [20]. Fennel oil is an essential oil extracted from *Foeniculum Vulgaris* [21]. Fennel oil possesses antimicrobial and anti-inflammatory properties, as found by Van Wyk and Wink [22]. Moreover, it has antioxidant [23,24,25], antimicrobial [26], and hepato-protective activities [27]. In rabbits, [28] revealed that intact fennel seeds as food additive improved growth rate and reduced total cholesterol. However, no other studies pointed to the effect of fennel oil on growth parameters, blood metabolites (creatinine, triglyceride (TG), alanine transaminase (ALT), aspartate transaminase (AST) and antioxidant parameters), and gene expression of apoptosis genes. In the current study, fennel essential oil was used as an antibiotic alternative compared to gentamycin for enhancing the expression of apoptosis genes and antioxidant enzymes in weaned rabbits as well as meat quality and growth performance.

## 2. Materials and Methods

### 2.1. Fennel Essential Oil Preparation and Characterization

Essential oil extract of fennel seeds (*Foeniculum vulgare* Mill.) was obtained from local market in Zagazig City, Egypt and evaluated using gas chromatography-mass spectrometry (GC-MS). The essential oil was analyzed by GC/MS on an HP-5MS capillary column covered with cross-linked methyl silicone gum (30 m × 0.25 mm diameter × 0.25 m thick). Helium was used as the carrier gas, with a flow rate of 1 ml/min. Temperature program: 35 °C held for 5 min, then heated to 260 °C at 10 °C each minute for 3 min. The injector had a temperature of 250 °C, whereas the MS interface had a temperature of 280 °C. A total of 0.1 μL of essential oil was injected. Chem Station software and the Wiley mass spectrum library were used to evaluate the results. Individual component relative percentages are expressed as percent peak area compared to the total composition of the EO as determined by GC-MS analysis [29].

### 2.2. Biological System, Experimental Design, and Environmental Data

According to the Local Experimental Animal Care Committee, the current experiment was carried out inside the Rabbits Research Unit, Agriculture college at Moshtohor, Benha University, Egypt. The ethics were approved by our local Institutional Committee. Animals have been raised according to the standards for husbandry of Benha University. A total of 45 Moshtohor (developed from crossing between V- line and Gabali concluded by Iraqi et al. [30]) four week-old weaned male rabbits with comparable average body weight (500 g) were randomly allocated into three treatments, each of which replicated five times with three animals per replicate (n = 15). Weaned rabbits were housed in cages (45 × 55 × 30 cm). The first treatment of rabbits was the control group, whereas the other two groups, T1 and T2, were orally supplied with 1 mL fennel oil and 1 mL gentamycin, respectively. The treatments have been applied twice weekly for the entire experimental period (8 weeks).

#### 2.2.1. Growth Performance

Weaned rabbits in each replicate were weighed at 4, 8, and 12 weeks of age, using a numerical scale, and the average daily weight gain (ADG (g/weaned rabbit)) was calculated. Five rabbits were taken randomly at the end of the experiment, from each treatment for all more experiments; the rabbits were slaughtered to evaluate internal organs’ carcass characteristics and weight. The carcass and inner organs were expressed in relation to the final body weight. In addition, rabbit Musculus Longissimus lumborum (LL) of each animal was dissected. The liver was prepared and immediately placed in liquid nitrogen and later frozen at −80 °C to assess antioxidant status, meat quality, and gene expression.

#### 2.2.2. Collection of Samples for Blood Hematological and Biochemical Parameters Evaluation

Five samples of rabbit blood from each treatment were gathered to measure the biochemical and hematological parameters. Blood samples from the two treatments and the control group were separated into two portions. The first portion was collected with 10% ethylene diamine tetraacetate (EDTA) as an anticoagulant to determine hematocrit (HCT), hemoglobin (Hgb), mean corpuscular volume (MCV), mean corpuscular hemoglobin (MCH), and mean corpuscular hemoglobin (MCHC), total red blood cell count (RBC), and total white blood cell count (WBC) by standard methods mentioned described by Jain [31]. The other portion was collected without anticoagulant, left to clot at 4 °C, then centrifuged at 3000 rpm for 10 min to recover the blood serum. The non-hemolyzed serum was collected and stored at −20 °C until the measurement of the biochemical parameters. Alanine transaminase (ALT) and aspartate transaminase (AST) were determined using the Morgenstern, Oklander [32] method. Serum levels of total cholesterol, triglyceride, and high-density lipoprotein cholesterol (HDL-C) were spectrophotometrically assessed using commercial kits developed by Pasteur laboratories (Egyptian American Co. for Laboratory Services, Giza, Egypt).

#### 2.2.3. Analysis of Meat Quality Parameters

Right and left Longissimus lumborum (LL) cuts were dissected from rabbits carcasses to determine pH, water holding capacity (WHC), drip loss (48 h), thawing, and cooking losses (samples taken 10 min to reach 75 °C in a preheated water bath), Warner-Bratzler Shear Force (WBSF), lightness (L *), redness (a *), yellowness (b *), chroma (C), and Hue angle (h) as described earlier [33]. Additionally, moisture contents were assessed by AOAC methodology [34]. The keeping quality of the rabbit’s meat was evaluated over 10 days, as stated previously for chicken [35], and rabbit meat [36] where the hind legs were directly separated from the rabbit carcasses under sterilized condition, the bone was trimmed, the meat portions from various replicates of the same group were minced together, and then 25 g of the meat homogenate were loaded into a sterilized 50 mL falcon tube. Three falcon tubes were distributed for each checkpoint (5 checkpoints; day 1, day 3, day 5, day 7, and day 10) and placed in a programmable incubator (Binder KB 23, BINDER GmbH (Headquarters), Tuttlingen, Germany) at 5 ± 0.2 °C for further determination of aerobic plate count (APC) and pH. APC was determined in the same manner as previously demonstrated for natural beef microflora [37], Briefly, a 10% meat homogenate was created by homogenizing a 10 g incubated sample with a 90 mL sterile peptone solution (0.1%). Then, using sterile normal saline, a serial 10-fold dilution of the sample’s homogenate was prepared, and the dilutions of each sample were inoculated in duplicate into APC agar. The plates were then incubated at 37 °C for 24–48 h before the colonies were counted. The pH was measured by the direct insertion of pH-meter glass electrode into minced meat within the falcon tube.

#### 2.2.4. Antioxidant Activities

To eliminate red blood clots, rabbit liver tissues were washed with phosphate-buffered saline (PBS) solution; pH 7.4 supplemented with 0.16 mg/mL heparin. The tissues were homogenized in 5 mL of cold PBS per gram of tissue (1:5 dilution). All those samples were centrifugated at 4000 rpm at 4 °C for 15 min. Supernatants were collected and stored at −20 °C until biochemical analysis of superoxide dismutase (SOD), catalase activity, malondialdehyde (MDA), and total antioxidant capacity (TAC) levels were carried out. TAC was estimated by the colorimetric method defined by Koracevic and Koracevic [38], the catalase enzyme was estimated by a method, as shown by Aebi [39], while the MDA was measured by the technique described by Ohkawa and Ohishi [40]. The Nishikimi and Rao [41] approach was followed for SOD analysis.

#### 2.2.5. RNA Isolation and cDNA Synthesis

For RNA isolation, tissue samples from the liver were collected from five rabbits in each treatment group and were flash-frozen with liquid nitrogen and then kept at −80 °C. According to the protocol, total RNA was isolated from liver tissue samples as described in Promega cat.no # Z3100. The cDNA synthesis was done from 1 µg of total RNA using a high-capacity cDNA reverse transcriptase (Thermo Fisher Scientific, Waltham, MA, USA). For each sample, the RT-PCR reactions in triplicate were performed, in addition to the non-template control (NTC) that was used to test the specificity and the purity of the primers and RNA samples, respectively. Real-time PCR reaction was performed on a Step One™ real-time system (applied biosystems) using Maxima SYBR Green/ROX qPCR master mix (2×) (Thermo Scientific, USA). The reaction mixture consisted of 2.5 μL of cDNA (excluding NTC), 12.5 μL of SYBR Green Master Mix, 0.75 μL of each forward and reverse primer pair, and 25 μL of Nucleases-Free Water. The PCR conditions were 95 °C for 10 min as a hot start, followed by 40 cycles of 95 °C for 15 s as a denaturation step, annealing/extension step at 60 °C for 1 min, and ending with a melt curve from 65 to 95 °C. Data were analyzed according to the 2^−ΔΔCt^ method [42] and were normalized by UXT expression in each sample. Changes in the expression rates of target genes were interpreted as n-fold changes in relation to the corresponding controls. The qRT-PCR primers used in this study are shown in Table 1.

#### 2.2.6. Quantitative Histomorphometric Analysis of Jejunum Segments

One segment (3 cm) of the mid-jejunum was collected from five rabbits from each treatment. Samples were applied to the formalin fixation for two days, then implanted into paraffin oil. Fixed samples were sectioned into (100 μm thick), two sections for each sample were taken, stained with hematoxylin for 1 min, then counterstained with eosin for 10 s, to measure the villus length (measurement was done from top of the crypt to the tip of the villus), villus width, number of villi in each section (NVIS), muscularis thickness (MTh), and goblet cells (G cell). All targeted variables were measured with a camera (OLYMPUS; TH4-200; Tokyo, Japan) coupled with computer-assisted digital-image pro plus (IPP) analysis software (Image-Pro Plus 4.5, Media Cybernetics, Silver Spring, MD, USA).

#### 2.2.7. Villus Morphology and Morphometry

Five segments of the mid-jejunum (3 cm) from each treatment were collected, fixed with formalin for 48 h, and paraffin-embedded. Two sections (100 μm) from each sample were obtained, stained with hematoxylin for 1 min, and counterstained with eosin for 10 s, to assess the maximum villus length (measured from above the crypt to the tip of the villus), villus width, goblet lining cells, and submucosa/muscularis/serosa thickness. All target variables were measured by a camera (OLYMPUS, TH4-200; Tokyo, Japan) and computer-aided digital image pro plus (IPP) analysis software (Image-Pro plus 4.5, Media Cybernetics, Silver Spring, MD, USA).

#### 2.2.8. Statistical Analysis

Data analysis was carried out using PROC GLM in SAS (1996) and expressed as mean value ± SEM. Rt-qPCR datasets were analyzed using PROC GLM of SAS [44]. Significant differences were considered at *p* <  0.05. Differences in the same group treatments were examined using Duncan’s multiple range test at *p* < 0.05. The static model applied is as follows:

yij = µ + Ti + eij

where y is the observations, µ is the general mean, Ti is the effect of treatment, and eij is the random error.

## 3. Results

### 3.1. Chemical Composition of Fennel Oil

Gas chromatography/mass spectrometry (GC–MS) was used to examine the chemical components of fennel essential oil. Table 2 shows the fennel essential oil constituents, retention time, and percentages. There was a total of 24 compounds discovered. EO had the highest amounts of volatile components (Anethole = 75%, Fenchone = 16%, α-a pinene = 4%, and limonene = 3.3%).

### 3.2. Growth Performance, Feed Intake Feed Conversion Ratio, and Carcass Traits

The results illustrated that adding fennel oil or gentamycin increased (*p* < 0.05) the BW at the 8 and 12 weeks and the average daily gain (ADG) from 4–8, 8–12, and 4–12 weeks compared to the non-treated group (the control group). There was a clear variance in feed conversion ratio (FCR) between the three groups during weeks 4 to 12 (Table 3). Weaned rabbits treated with fennel oil or gentamycin grew 23.02, 28.78 and 25.37% for the fennel oil group and 20.4, 35.87 and 27.57%, for gentamycin group, faster compared to the rabbits in the control group from week 4–8, 8–12, and 4–12, respectively (Table 3). The supplementation of fennel oil or gentamycin showed no statistically significant effects on carcass cut weights and internal organs except for head rate (Table 4).

### 3.3. Physical Characteristics and Microbial Abundance of MLD Muscle

Table 5 showed that current treatment had no effect (*p* > 0.05) on WHC, drip loss (48 h), lightness (L*), thawing loss, cooking loss, redness (a*) and yellowness (b*), and Hue angle (h) of *Longissimus lumborum* (LL), but there were substantial pH differences (*p* < 0.05). *Longissimus lumborum* (LL) meat from fennel and gentamycin also displayed higher shear force and lower moisture content than the control (*p* < 0.05). In addition, chroma (c) value was higher in gentamycin served rabbits (*p* < 0.05). Regarding keeping quality assessment, the initial APC (day 1) did not vary between comparable groups (*p* > 0.05), but the pH values observed were different at most checkpoints and all were lower than control rabbits’ pH (*p* < 0.05). On the third day of chilling, fennel oil-treated rabbits and the control group produced higher APC and pH values (*p* < 0.05) than the gentamycin-treated rabbits. On the fifth day of chilling, rabbits treated with fennel oil and gentamycin revealed the lowest pH values (*p* < 0.05) compared to the control group. On the seventh day of chilling test, there were marked differences in APC between the different groups, where rabbits treated with fennel oil displayed lower microflora counts than the control and gentamycin groups (*p* < 0.05). On the tenth day of carcass chilling, both treatments (fennel oil and gentamycin) had a similar pH, which was statistically lower than the control group. On the tenth day of chilling, it was also observed that only fennel oil and gentamycin-treated groups still hold natural microflora counts lower than control. The pH values for the last two checkpoints, day 7 and day 10, also ranged from group to group (*p* < 0.05).

### 3.4. Blood Hematological and Biochemical

The consequences of additives on hematological variables of blood are listed in Table 6. There were no statistically significant differences in hematological variables among the treatment groups except for RBCs, hematocrit, MCV, and eosinophils. Weaning rabbits supplemented with gentamycin exhibited lower RBC count and higher MCV than those of the control group. Meanwhile, weaning rabbits supplemented with fennel oil or gentamycin had a significantly lower hematocrit and eosinophils percentage than the controls. As shown in Table 7, there were no significant differences between control and treated groups on kidney and liver functions except for AST. Generally, weaning rabbits supplemented with fennel oil or gentamycin had decreased total cholesterol and triglyceride than the control weaning rabbits. Extra fennel oil had a significant increase in the concentration of HDL.

### 3.5. Antioxidant Enzyme Activity

The antioxidant markers of weaned rabbits as they were influenced by the supplementation are demonstrated in Table 8. In contrast to the control group, SOD, CAT, and TAC concentrations were increased at week 12 in treated weaned rabbits. Meanwhile, the concentration of MDA was contrary to those observed in SOD, CAT, and TAC.

### 3.6. Villus Morphology and Morphometry

Data regarding intestinal morphology of weaned Moshtohor rabbits aged 12 weeks are shown in Table 9 and Figure S1. Although the NVIS was consistently higher in the additives group, it was not statistically significant. However, the weaned rabbit which received fennel oil or gentamycin as a supplement had a higher villus width and villus length than those in the control group. Furthermore, the supplementation of fennel oil or gentamycin improved the MTh and G cells in the weaned rabbit.

### 3.7. Antioxidant and Apoptosis Genes Expression

**Figure 1:**
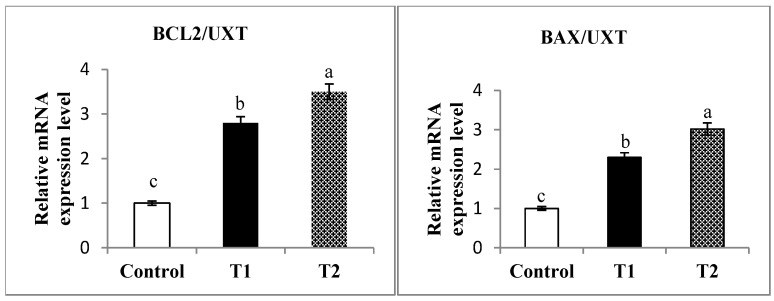

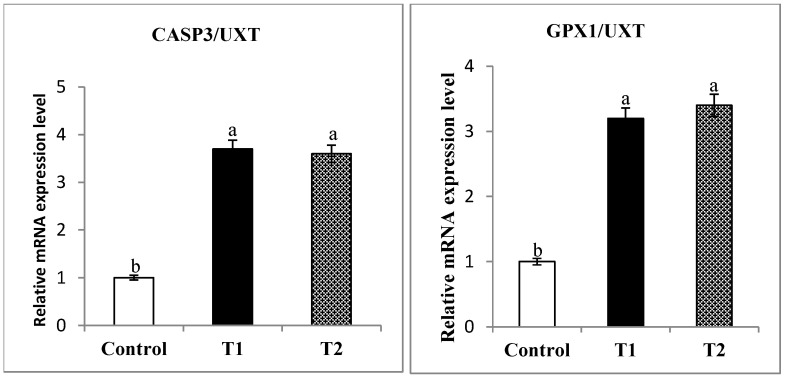
The qRT-PCR validation of mRNA expression for *BAX/UXT, BCL2/UXT, CASP3/UXT,* and *GPX1/UXT* in liver tissue; cell lymphoma2: *BAX/UXT+ BCL2/UXT*; caspase: *CASP3/UXT*; glutathione peroxidase: *GPX1/UXT*. ^a,b,c^ values in the same box with a different superscript differ significantly at *p* < 0.05.

The results of relative mRNA expression levels in the liver tissue are presented in Figure 1. The mRNA expression of BAX and CASP3 was downregulated. Meanwhile, BCL2 and GPX1 were upregulated in the fennel oil and gentamycin treatment groups compared to the control group.

## 4. Discussion

Fennel oil is an essential oil that plays a vital role in promoting the digestion phase by enhancing digestive fluids’ secretion, enzyme stimulation, and reducing the effects of pathogenic bacteria. Although the GC-MS data detected 24 compounds, the principal compounds accounted for 98.3% of the total compounds identified. Despite the fact that information on the biological activity of majority of the discovered compounds is insufficient, antioxidative and antibacterial properties have been described [45]. The good biological activities of the additive’s mixture may be attributed to the positive effects of feeding them in the current assay. The antimicrobial and antioxidant activity of the fennel oil may be due to the chemical composition of EO. Anwar et al. [45] recorded that anethole, the main component of fennel essential oil, showed antioxidant, antibacterial, and antifungal activities. The majority of the literature has focused on the effect of fennel seeds on rabbit growth and cecal microflora. In this study, we focused on the influence of fennel oil on rabbit growth, blood metabolite, and gene expression of some selective apoptosis genes. The present study showed that the essential fennel oil or antibiotic alone could positively affect the growth performance of weaned rabbits. There is minimal information on the effects of fennel oil and gentamycin on the growth of rabbits. Ertas et al. [46] showed that ADWG of birds is improved by a mix of essential oils in diets compared to the antibiotic group, whereas Benlemlih et al. [47] stated that supplementation of the weaned rabbit with fennel oil, thyme oil, and oxytetracycline did not affect growth performance. Diet supplementation of weaned rabbits with fennel seeds led to an improvement in body weight [28]. Al-Kassie et al. [48] showed that the inclusion of a mixture of anise and rosemary at the rate of 0.5 to 1% increased the growth and improved feed conversion ratio related to its active compounded such as anethole, borneol, and carnosic acid. Schöne et al. [49] showed the major fennel oil compound was anethol, representing 50–70%. Some experiments have suggested that such components led to promoting growth performance [50], which may be due to the activated enzymes that improve the digestive process in the body [51]. Fennel essential oil reduced cholesterol levels in rabbits, indicating a positive modulatory effect on cholesterol metabolism and turnover, tissue protection against lipid peroxidation, and considerable lipid-lowering efficacy. These results back up the additive supplementation’s cardiovascular beneficial effects. Because the additives mixture may contain phenolic compounds that elicit antioxidant action by scavenging reactive oxygen species, enhancing cellular antioxidant enzymes (e.g., superoxide dismutase, catalase, and glutathione peroxidase), and increasing glutathione, the fennel essential oil increased HDL cholesterol and triglycerides and decreased MDA [52].

In addition, our results illustrate that the antibiotic and fennel oil increase the villi length and mucus thickness (MTh) in weaned rabbits and, therefore, may increase nutrient absorption and greater growth performance. This result agrees with the findings of Hernandez et al. [53], who found that the addition of essential oils perfected apparent total tract and ileal digestibility of nutrients. Additionally, Coelho-de-Souza et al. [54] reported that the essential oil of *Croton zehntneri* and anethole was able to enhance the gastric wall mucus yield, an essential gastro-protective factor.

Little information on the effects of dietary supplementation of essential oils, including fennel oil, on rabbit meat quality is known, and more in-depth investigations are required [55]. In the current study, dietary fennel supplementation did not alter rabbit meat’s technological quality attributes and primary color parameters (L*, a*, b*, and Hue). Similar findings were recorded with pork and heifers when the oleoresins essential oils of rosemary, garlic, oregano or ginger, and clove were used [55,56,57]. Nevertheless, the reduction in rabbit meat pH was correlated with groups treated with fennel oil and antibiotics. This may indicate that fennel and antibiotic supplementation has increased the glycolytic metabolism of the LL and hind leg muscles, particularly in comparison to control. Ranucci et al. Ranucci, Beghelli [56] also found that the pH values for pork-fed essential oil were less than the untreated group, but controversial results were documented in another report [55,57]. Besides that, the low moisture content of gentamycin and fennel may explain the high force needed to shear cooked meat derived from them and indicates tougher cuts. Current changes in tenderness and chroma have not been reported earlier with pork and feedlot heifers [55,57]. Despite the fact that the current instrumental color L*, a*, and h° values were not statistically significant between groups, they indicate that antibiotic-served rabbit meat will be redder than all other groups, and fennel-supplied rabbit produced brighter red meat than the control [58]. The shelf-life evaluation showed that fennel had satisfied antimicrobial properties greater than the antibiotic-treated group and to the point that fennel oil could inhibit APC on the 7th day compared to other groups. However, after 10 days of chilling at 5 °C, all meat derived from rabbits treated with fennel and antibiotic containing diets reached the critical threshold of spoilage APC for fresh meat, 6–7 log CFU/g. It was reported earlier that after 5 days of storage at 3 °C, most of the rabbit carcasses had APC of about 7 log CFU/g and reached 8 logs CFU/g after 6 to 8 days [59]. In addition to strong antioxidant criteria due to the high content of polyphenols and flavonoids [43,60], this finding suggests that feeding fennel oil can potentially substitute antibiotics to improve the shelf-life of rabbit meat. These properties have also been widely reported for many other essential oils and their constituents [61]. The results reflecting the improved quality of rabbit meat obtained after feeding on the enriched diets are consistent with previous studies [36,62].

In this study, the addition of different additives decreased serum MDA levels, increased SOD, CAT, and TAC concentration. The concentration of MDA is considered one of the most important biomarkers for lipid peroxidation. However, SOD and CAT are beneficial enzymes since they play a vital role in scavenging the free radicals such as superoxide anion or hydrogen peroxide from the cells [63]. It has been reported that fennel oil acts as an antioxidant because of its ability to inhibit lipid peroxidation [64]. In this study, the higher SOD, CAT, and TAC showed in the fennel oil and gentamycin groups may have shown an increase in SOD, CAT, and TAC suggesting that fennel oil and gentamycin showed a reduction of oxidative stress in the treated animals and improved health as revealed in increasing BW.

Nutrition is a crucial aspect of rabbit production, affecting blood metabolites [65,66]. In our study, the observed decrease of cholesterol in supplemented groups compared to controls may probably be due to an inhibition of the hepatic 3-hydroxy-3-methylglutaryl coenzyme A reductase activity, which promotes the synthesis of cholesterol [67]. Case et al. [68] demonstrated that ether thymol extracts reduced blood triglyceride concentrations in Leghorn chickens, which may be associated with its active compound and decrease the lipolysis enzymes activity, thus decreasing hepatic fatty acid synthesis. Sedláková et al. [69] stated that the presence of thymol, thymosin, anethole, and carvone in fennel and cumin plants reduced the level of cholesterol and triglyceride which may be associated with a reduction in fat uptake in the intestines. HDL exhibits a wide range of anti-thermogenic effects such as antioxidant and anti-inflammatory activities and enhancing the cholesterol efflux, which affects the formation of cell foam and reverses cholesterol transport [70,71]. Shahidullah et al. [72] showed that gentamycin supplementation in rats increased serum HDL compared to controls. Additionally, Hong et al. [73] reported that broilers supplemented with an essential oil such as oregano, anise, and citrus peel had higher HDL levels than the controls. According to our data, the higher level of HDL and lower concentrations of total cholesterol and triglyceride suggested that the fennel oil could have a positive regulatory effect on lipid metabolism in weaned rabbits.

ALT and AST have been used as an indicator for liver normality [74,75]. The decrease in liver function can hypothesize better liver function [76,77]. Kumar and Nazir et al. [78,79] have reported that *Funiculus vulgare* reduces ALT levels, alkaline phosphatase (ALP), and AST in the serum. According to our data, the fennel oil and gentamycin supplementation significantly decreased AST, improving liver function. According to Bovera et al. [80] and Moniello et al. [81], AST can be found in many tissues such as muscles. Therefore, it is known to be a marker for liver function. In the current study, the effect of gentamycin and fennel oil on hematocrit was parallel with the RBC count, indicating a decrease in the amount of oxygen received by tissues. Additionally, gentamycin treatment-induced changes in MCV were attributed to a direct response to the low RBC count. Essential oils act as anti-inflammatory agents [82]. Zhang et al. [83] reported that the main component of the essential fennel oil, trans-anethole, possesses anti-inflammatory and antibacterial properties. Our results also showed that after fennel oil supplementation, inflammatory cells (eosinophils) were reduced in weaned rabbits.

The obtained outcomes in this study demonstrated that the relative mRNA expression for BAX, Caspase3, BCL2, and GPX1 in different treatments was down and upregulated, respectively, compared to controls. BAX and BCL2 genes are involved in BCL2 gene families that act as pro-apoptotic [84] and anti-apoptotic [85], respectively. Caspase-3 is one of the major effectors of apoptosis, and activation of caspase-3 indicates irreversible cell apoptosis [86]. Apoptosis is a biological mechanism for removing dysfunctional cells. Thus apoptosis dysregulation is thought to enhance pathogenesis progression [84]. Okuno et al. [87] demonstrated that overexpression of Bcl-2 can inhibit caspase-independent apoptosis. It is plausible that decreased CASP3 mRNA expression in different supplemented groups may be due to increased Bcl-2, which is involved in apoptosis in supplemented groups. This result indicates that Bax has a pro-apoptotic function in cells [88], whereas Bcl-2 exhibits anti-apoptotic function [89]. It has been reported that the apoptosis rate is inversely proportional to the Bcl-2 expression level [90]. This may suggest that up-regulation of BCL-2 plays a role in rabbit cell differentiation and removes dysfunctional cells. Glutathione peroxidase (GPX1) is an antioxidant gene increased due to the supplementation of essential oil or antibiotics. It was established that GPX1 reduces and detoxifies the hydrogen peroxide and organic hydroperoxides [91]. Thus, the up-regulation of this gene on the different groups could justify the reduction of the lipid peroxidation products in our study and reduce oxidative stress damages.

## 5. Conclusions

Under the conditions of the present study, supplementation of weaned Moshtohor rabbits with fennel oil acts as a natural alternative for gentamycin enhanced feed conversion and daily gain through enhancing villus length and mucus thickness. Additionally, fennel essential oil reduces the oxidative stress by increasing the antioxidant enzymes. Finally, the current study demonstrated that fennel oil could be used as an antibiotic alternative to improve growth performance, meat shelf-life, and reduce oxidative stress in rabbits.

## Figures and Tables

**Table 1:** Primers used for quantitative real-time PCR analysis of gene expression according to Imbabi et al. [43].

Gene	Accession Number	Primers Sequences (5′→3′)	Product Size (bp)
*UXT*	XM_008272555	F: GCGGGACTTGCGAAAGGT	100
		R: AGCTTCCTGGAGTCGTTCAATG	
*BAX*	XM_008252361.2	F: CCCGCGAGGTCTTTTTCC	113
		R: CAGGGCCTTGAGTACCAGCTT	
*Bcl-2*	XM_008261439.2	F: GGCTGGGATGCCTTCGT	186
		R: TTTCGTGAACTGTTTGCATATCTG	
*CASP3*	NM_008261439.2	F: GACAGTGGCATCGAGACAGACA	110
		R: GAATAGTAACCAGGTGCTGTGGAA	
*GPX1*	NM_001085444.1	F: CAGTTTGGGCATCAGGAGAAC	94

UXT: Ubiquitously expressed prefoldin like chaperone; BAX: BCL2 Associated X, Apoptosis Regulator; Bcl-2: B-cell lymphoma 2; CASP3: Cysteine-aspartic proteases; GPX1: Glutathione peroxidase 1.

**Table 2:** Major component in fennel essential oil.

Retention Time (min)	Component	Concentration (%)
5	α-a pinene	4
7.6	limonene	3.3
11	Fenchone	16
16.7	Anethole	75

**Table 3:** Impact of dietary fennel oil or gentamycin on growth performance, feed intake, and feed conversion ratio of weaning Moshtohor rabbits aged 4–12 weeks.

Growth Parameter	Control	Fennel	Gentamycin	SEM	*p* Value
Body weight (BW) (g)					
BW4	501.0	501.7	499.3	1.69	0.6262
BW8	1007.3 ^b^	1124.0 ^a^	1110.0 ^a^	5.67	0.0001
BW12	1443.0 ^b^	1684.7 ^a^	1700.6 ^a^	36.28	0.0040
Average daily gain (ADG)(g/d)					
ADG8-4	18.07 ^b^	22.23 ^a^	21.77 ^a^	0.208	0.0001
ADG12-8	15.53 ^b^	20.00 ^a^	21.10 ^a^	1.261	0.0445
ADG12-4	16.83 ^b^	21.10 ^a^	21.47 ^a^	0.643	0.0039
Feed intake (FI) (g)					
FI 4-12	70.60	71.00	71.47	0.345	0.3468
Feed conversion ratio (g/g)					
FCR 4-12	2.75 ^a^	2.36 ^b^	2.35 ^b^	0.063	0.0067

All data are expressed as the mean with SEM in same row; ^a,b^ letters indicate significant differences between means (*p* < 0.05). BW4: initial body weight at four weeks; BW8: body weight at eight weeks; BW12: final body weight at 12 weeks; ADG8-4: average daily gain from 4 to 8 weeks; ADG8-12: average daily gain from 8 to 12 weeks; ADG4-12: average daily gain from 4 to 12 weeks.

**Table 4:** Impact of dietary fennel oil or gentamycin on the relative weights of carcass cuts and internal organs of weaned Moshtohor rabbits aged 12 weeks.

Parameters	Control	Fennel	Gentamycin	SEM	*p* Value
Carcass cuts					
Live body weight (g)	1443.00 ^b^	1684.67 ^a^	1700.67 ^a^	36.28	0.0004
Carcass (%)	51.20	45.06	47.03	2.43	0.2656
Head rate (%)	7.29 ^a^	6.20 ^a,b^	5.52 ^b^	0.356	0.0336
Hind legs rate (%)	14.03	13.11	15.33	1.034	0.3730
Saddle rate (%)	10.33	9.57	10.69	0.414	0.2269
Fore legs rate (%)	10.20	9.73	10.25	0.239	0.3155
Thoracical neck rate (%)	12.42	9.65	13.57	1.254	0.1546
Body organs (%)					
Liver (%)	2.86	2.70	2.38	0.169	0.2125
Kidney (%)	0.672	0.584	0.559	0.047	0.2832
Spleen (%)	0.074	0.069	0.049	0.009	0.1949
Lung (%)	0.739	0.613	0.676	0.068	0.4748
Heart (%)	0.309	0.267	0.235	0.021	0.1252

All data are expressed as the mean with SEM in same row; ^a,b^ letters indicate significant differences between means (*p* < 0.05). Rabbit sample/group, *n* = 5. The parameters rate and organ index were calculated as follows: parameters rate or organ index = (organ weight/living weight) × 100%.

**Table 5:** Impact of dietary fennel oil or gentamycin on meat quality of weaned Moshtohor rabbits aged 12 weeks.

Meat Quality	Control	Treatment		SEM	*p*-Value
		Fennel	Gentamycin		
pH (24 h)	6.00 ^a^	5.86 ^b^	5.82 ^b^	0.023	0.000
WHC	85.75	83.36	84.71	2.819	0.726
Drip loss (48 h) %	1.50	1.13	1.12	0.187	0.667
Thawing loss	7.26	6.45	10.01	1.365	0.431
Cooking loss %	16.89	20.59	17.46	1.256	0.284
WBSF	4.12 ^c^	5.44 ^b^	6.68 ^a^	0.25	0.0001
L*	54.59	54.64	51.89	0.69	0.24
a*	11.45	11.90	13.03	0.84	0.72
b*	6.23	5.68	5.77	0.19	0.08
C	13.04 ^b^	13.19 ^b^	14.25 ^a^	0.30	0.03
h°	28.46	25.57	23.88	0.84	0.05
Moisture	74.39 ^a^	71.17 ^b^	69.50 ^b^	0.336	0.009
Keeping quality test					
APC (log CFU/g)					
Day 1	3.76	4.08	3.85	0.165	0.575
Day 3	4.58 ^b^	4.71 ^a^	4.45 ^c^	0.014	0.003
Day 5	5.24	4.90	5.06	0.08	0.050
Day 7	5.67 ^a^	5.03 ^b^	5.57 ^a^	0.06	0.009
Day 10	6.22 ^b^	6.94 ^a^	6.85 ^a^	0.18	0.018
pH					
Day 1	5.98 ^a^	5.90 ^b^	5.83 ^c^	0.013	0.000
Day 3	5.86 ^a^	5.77 ^b^	5.69 ^c^	0.014	0.000
Day 5	6.06 ^a^	6.01 ^b^	5.84 ^d^	0.006	0.000
Day 7	6.03 ^a^	6.04 ^a^	5.87 ^b^	0.006	0.000
Day 10	6.10 ^a^	5.95 ^b^	5.91 ^b^	0.017	0.003

All data are expressed as the mean with SEM in same row; ^a,b,c^ letters indicate significant differences between means (*p* < 0.05).

**Table 6:** Impact of dietary fennel oil or gentamycin on hematological variable of blood serum of weaned Moshtohor rabbits aged 12 weeks.

Parameters	Control	Fennel	Gentamycin	SEM	*p* Value
Hematological variable of blood					
Hemoglobin (g/dL)	20.00	18.60	17.97	0.620	0.1374
RBCs (10^6/cmm)	7.50 ^a^	6.80 ^a^	5.95 ^b^	0.214	0.0068
HTC (vol%)	46.00 ^a^	41.50 ^b^	43.00 ^a,b^	0.897	0.0313
MCV (fl)	61.60 ^b^	60.97 ^b^	72.30 ^a^	1.568	0.0036
MCH (pg)	26.70	27.50	30.17	1.107	0.1467
MCHC (%)	43.50	45.07	41.83	2.134	0.5914
Platelets (10^3/cmm)	644.5	710.0	683.5	63.09	0.7702
WBC (10^3/cmm)	10.10	9.97	10.20	0.731	0.9748
Neutrophils %	68.50	71.00	69.00	1.12	0.3170
Lymphocytes %	24.00	25.00	24.50	1.26	0.8574
Monocytes%	6.00	3.50	4.50	0.624	0.0764
Eosinophils %	1.50 ^a^	0.500 ^b^	2.00 ^a^	0.236	0.0110
Basophils %	0	0	0	0	0

^a,b^ values in the same row with a different superscript differ significantly at *p* < 0.05. RBCs = red blood cell count, HTC = hematocrit, WBC = white blood cell count, MCV = mean corpuscular volume, MCH= mean corpuscular hemoglobin, MCHC= mean corpuscular hemoglobin concentration.

**Table 7:** Impact of dietary fennel oil or gentamycin on blood biochemical parameters of weaned Moshtohor rabbits aged 12 weeks.

Parameter	Control	Fennel	Gentamycin	SEM	*p* Value
AST (U/I)	49.76 ^a^	33.65 ^b^	26.19 ^b^	2.244	0.0008
ALT (U/I)	48.02	58.49	57.62	11.14	0.7707
Creatinine (mg/dL)	2.32	2.18	2.56	0.094	0.0762
Total Cholesterol (mg/dL)	80.59 ^a^	64.48 ^b^	54.11 ^c^	2.921	0.0020
High-density lipoprotein (mg/dL)	43.92 ^b^	57.11 ^a^	46.98 ^b^	2.222	0.0133
Triglyceride (mg/dL)	27.29 ^a^	23.87 ^b^	20.08 ^c^	0.576	0.0004

^a,b,c^ values in the same row with a different superscript differ significantly at *p* < 0.05.

**Table 8:** Impact of dietary fennel oil or gentamycin on the antioxidant parameter in liver tissue of weaned Moshtohor rabbits aged 12 weeks.

Antioxidant Parameter	Control	Fennel Oil	Gentamycin	SEM	*p* Value
MDA (nM/g)	265.42 ^a^	238.96 ^b^	217.20 ^c^	3.240	0.0001
SOD (Ul/g)	151.73 ^b^	165.63 ^b^	235.31 ^a^	5.452	0.0001
CAT (u/mg)	3.36 ^b^	3.90 ^a^	3.99 ^a^	0.101	0.0091
TAC (mM/g)	9.76 ^b^	13.37 ^a^	13.37 ^a^	0.229	0.0001

All data are expressed as the mean with SEM in same row; ^a,b,c^ letters indicate significant differences between means (*p* < 0.05). Rabbit sample/group *n* = 5. MDA: Malondialdehyde; CAT: catalase; SOD: superoxide dismutase; TAC: total antioxidant capacity.

**Table 9:** Impact of dietary fennel oil or gentamycin on morphology of intestinal weaned Moshtohor rabbits aged 12 weeks.

Parameters	Control	Fennel Oil	Gentamycin	SEM	*p* Value
NVIS (100 μm)	47.11	54.00	56.00	4.789	0.4016
Villus width (100 μm)	104.00	128.00	134.00	9.695	0.0890
Villus length (100 μm)	351.11 ^b^	477.00 ^a^	526.44 ^a^	19.14	0.0001
MTh (100 μm)	64.00 ^b^	77.00 ^a,b^	89.00 ^a^	5.123	0.0079
G cell (100 μm)	16.76	18.30	19.65	1.424	0.7434

^a,b^ values in the same row with a different superscript differ significantly at *p* < 0.05. NVIS = number of villi in section, MTh = muscularis thickness, G cell = goblet cells.

## Data Availability

Data is contained in the manuscript and supplementary materials.

## Supplementary Materials

The following are available online at https://www.mdpi.com/article/10.3390/antiox10111797/s1, Supplementary Figure S1. Villus morphology and morphometry characteristics of the small intestine in the rabbits receiving fennel oil and Gentamycin.

## References

[1] Bhatt R.S., Agrawal A.R., Sahoo A. (2017). Effect of probiotic supplementation on growth performance, nutrient utilization and carcass characteristics of growing *Chinchilla rabbits*. J. Appl. Anim. Res..

[2] Cesari V., Toschi I., Ferrazzi V., Cesari N., Grilli G., Lavazza A. (2007). Effect of weaning age and diet on growth performance, caecal characteristics and potential pathogenetic microflora in rabbits. Ital. J. Anim. Sci..

[3] Ali B. (2003). Agents ameliorating or augmenting experimental gentamicin nephrotoxicity: Some recent research. Food Chem. Toxicol..

[4] Khaki A., Novin M.G., Khaki A.A., Nouri M., Sanati E., Nikmanesh M. (2008). Comparative Study of the Effects of Gentamicin, Neomycin, Streptomycin and Ofloxacin Antibiotics on Sperm Parameters and Testis Apoptosis in Rats. Pak. J. Biol. Sci..

[5] Falcão-e-Cunha L., Castro-Solla L., Maertens L., Marounek M., Pinheiro V., Freire J., Mourão J.L. (2007). Alternatives to antibiotic growth promoters in rabbit feeding: A review. World Rabbit. Sci..

[6] Maertens L.L.C. (2011). Strategies to reduce antibiotic use in rabbit production. J. Agric. Sci. Technol..

[7] Behrooz Lak M.A., Hassan Abadi A., Nasiri Moghadam H., Kermanshahi H. (2014). Effect of different levels of Cinnamon Powder, with Antibiotic and Probiotic on Performance and Carcass characteristics of Broiler Chickens. Res. Anim. Prod..

[8] Khan R., Naz S., Nikousefat Z., Tufarelli V., Laudadio V. (2012). Thymus vulgaris: Alternative to antibiotics in poultry feed. World’s Poult. Sci. J..

[9] Ashour E.A., El-Hack M.E.A., Alagawany M., Swelum A.A., Osman A.O., Saadeldin I., Abdel-Hamid M., Hussein E.-S.O. (2019). Use of Whey Protein Concentrates in Broiler Diets. J. Appl. Poult. Res..

[10] Kishawy A.T.Y., Amer S.A., Osman A., Elsayed S.A.M., El-Hack M.E.A., Swelum A.A., Ba-Awadh H., Saadeldin I.M. (2018). Impacts of supplementing growing rabbit diets with whey powder and citric acid on growth performance, nutrient digestibility, meat and bone analysis, and gut health. AMB Express.

[11] Omar A.E., Al-Khalaifah H.S., Mohamed W.A.M., Gharib H.S.A., Osman A., Al-Gabri N.A., Amer S. (2020). Effects of Phenolic-Rich Onion (*Allium cepa* L.) Extract on the Growth Performance, Behavior, Intestinal Histology, Amino Acid Digestibility, Antioxidant Activity, and the Immune Status of Broiler Chickens. Front. Veter Sci..

[12] Osman A., Bin-Jumah M., El-Hack M.E.A., Elaraby G., Swelum A.A., Taha A.E., Sitohy M., Allam A., Ashour E.A. (2020). Dietary supplementation of soybean glycinin can alter the growth, carcass traits, blood biochemical indices, and meat quality of broilers. Poult. Sci..

[13] Amer S., Ahmed S.A., Ibrahim R.E., Al-Gabri N.A., Osman A., Sitohy M. (2020). Impact of partial substitution of fish meal by methylated soy protein isolates on the nutritional, immunological, and health aspects of *Nile tilapia*, *Oreochromis niloticus* fingerlings. Aquaculture.

[14] El-Araby D.A., Amer S.A., Attia G.A., Osman A., Fahmy E.M., Altohamy D.E., Alkafafy M., Elakkad H.A., Tolba S.A. (2021). Dietary *Spirulina platensis* phycocyanin improves growth, tissue histoarchitecture, and immune responses, with modulating immunoexpression of CD3 and CD20 in *Nile tilapia*, *Oreochromis niloticus*. Aquaculture.

[15] Amer S.A., Osman A., Al-Gabri N.A., Elsayed S.A.M., El-Rahman G.I.A., Elabbasy M.T., Ahmed S.A.A., Ibrahim R.E. (2019). The Effect of Dietary Replacement of Fish Meal with Whey Protein Concentrate on the Growth Performance, Fish Health, and Immune Status of *Nile tilapia* Fingerlings, *Oreochromis niloticus*. Animals.

[16] Zeng Z., Zhang S., Wang H., Piao X. (2015). Essential oil and aromatic plants as feed additives in non-ruminant nutrition: A review. J. Anim. Sci. Biotechnol..

[17] Yang C., Chowdhury M.A.K., Huo Y., Gong J. (2015). Phytogenic Compounds as Alternatives to In-Feed Antibiotics: Potentials and Challenges in Application. Pathogens.

[18] Stoni A., Zitterl-Egelseer K., Kroismayr A., Wetscherek W., Windisch W. (2006). Tissue recovery of essential oils used as feed additive in piglet feeding and impact on nutrient digestibility. Proc. Soc. Nutr. Physiol..

[19] Stevanović Z.D., Bošnjak-Neumüller J., Pajić-Lijaković I., Raj J., Vasiljević M. (2018). Essential Oils as Feed Additives—Future Perspectives. Molecules.

[20] Calo J.R., Crandall P.G., O’Bryan C.A., Ricke S.C. (2015). Essential oils as antimicrobials in food systems—A review. Food Control..

[21] Hammouda F., Saleh M., Abdel-Azim N., Shams K., Ismail S., Shahat A., Saleh I. (2014). Evaluation Of The Essential Oil Of *Foeniculum Vulgare* Mill (Fennel) Fruits Extracted By Three Different Extraction Methods By Gc/Ms. Afr. J. Tradit. Complement. Altern. Med..

[22] Van Wyk B.E., Wink M. (2018). Medicinal Plants of the World.

[23] Roby M., Sarhan M.A., Selim K.A.-H., Khalel K.I. (2013). Antioxidant and antimicrobial activities of essential oil and extracts of fennel (*Foeniculum Vulgare* L.) and chamomile (*Matricaria chamomilla* L.). Ind. Crop. Prod..

[24] El Ouariachi E., Lahhit N., Bouyanzer A., Hammouti B., Paolini J., Majidi L., Desjobert J.M., Costa J. (2014). Chemical composition and antioxidant activity of essential oils and solvent extracts of *Foeniculum Vulgare* Mill. from Morocco. J. Chem. Pharm. Res..

[25] Hassaan M.S., Soltan M. (2016). Evaluation of Essential Oil of Fennel and Garlic Separately or Combined with *Bacillus licheniformis* on the Growth, Feeding Behaviour, Hemato-biochemical Indices of *Oreochromis niloticus* (L.) Fry. J. Aquac. Res. Dev..

[26] Shahat A., Ibrahim A.Y., Hendawy S.F., Omer E., Hammouda F., Abdel-Rahman F.H., Saleh M.A. (2011). Chemical Composition, Antimicrobial and Antioxidant Activities of Essential Oils from Organically Cultivated Fennel Cultivars. Molecules.

[27] Rather M.A., Dar B.A., Sofi S.N., Bhat B.A., Qurishi M.A. (2016). *Foeniculum Vulgare*: A comprehensive review of its traditional use, phytochemistry, pharmacology, and safety. Arab. J. Chem..

[28] Omer H.A., El-Nomeary Y.A., El-Kady R.I., Badr A.M., Ali F.A., Ahmed S.M., El-Allawy H.M., Ibrahim S.A. (2013). Improving the utilization of rabbit diets containing vegetable oil by using fennel (*Foeniculum Vulgare*) and oregano (*Origanum vulgare* L.) as feed additives. Life Sci. J..

[29] Mandras N., Roana J., Scalas D., Del Re S., Cavallo L., Ghisetti V., Tullio V. (2021). The Inhibition of Non-*albicans Candida* Species and Uncommon Yeast Pathogens by Selected Essential Oils and Their Major Compounds. Molecules.

[30] Iraqi M., García M., Khalil M., Baselga M. (2010). Evaluation of milk yield and some related maternal traits in a crossbreeding project of Egyptian Gabali breed with Spanish V-line in rabbits. J. Anim. Breed. Genet..

[31] Jain N.C. (1983). Hematological techniques. Schalm’s Veterinary Hematology.

[32] Morgenstern S., Oklander M., Auerbach J., Kaufman J., Klein B. (1966). Automated Determination of Serum Glutamic Oxaloacetic Transaminase. Clin. Chem..

[33] Elokil A.A., Imbabi T.A., Mohamed H.I., Abouelezz K.F.M., Ahmed-Farid O., Shishay G., Sabike I.I., Liu H. (2019). Zinc and Copper with New Triazine Hydrazone Ligand: Two Novel Organic Complexes Enhanced Expression of Peptide Growth Factors and Cytokine Genes in Weaned V-Line Rabbit. Animals.

[34] Horwitz W. (2000). Official Methods of Analysis of AOAC International.

[35] El-Bahr S., Shousha S., Shehab A., Khattab W., Ahmed-Farid O., Sabike I., El-Garhy O., Albokhadaim I., Albosadah K. (2020). Effect of Dietary Microalgae on Growth Performance, Profiles of Amino and Fatty Acids, Antioxidant Status, and Meat Quality of Broiler Chickens. Animals.

[36] Osman A., Imbabi T., El-Hadary A., Sabeq I., Edris S., Merwad A.-R., Azab E., Gobouri A., Mohammadein A., Sitohy M. (2021). Health Aspects, Growth Performance, and Meat Quality of Rabbits Receiving Diets Supplemented with Lettuce Fertilized with Whey Protein Hydrolysate Substituting Nitrate. Biomolecules.

[37] Sabike I., Fujikawa H., Edris A.M. (2015). The Growth Kinetics of Salmonella Enteritidis in Raw Ground Beef. Biocontrol Sci..

[38] Koracevic D., Harris G., Rayner A., Blair J., Watt B. (2001). Method for the measurement of antioxidant activity in human fluids. J. Clin. Pathol..

[39] Aebi H. (1984). Catalase in vitro. Methods in Enzymology.

[40] Ohkawa H., Ohishi N., Yagi K. (1979). Assay for lipid peroxides in animal tissues by thiobarbituric acid reaction. Anal. Biochem..

[41] Nishikimi M., Rao N.A., Yagi K. (1972). The occurrence of superoxide anion in the reaction of reduced phenazine methosulfate and molecular oxygen. Biochem. Biophys. Res. Commun..

[42] Livak K.J., Schmittgen T.D. (2001). Analysis of relative gene expression data using real-time quantitative PCR and the 2^−ΔΔCT^ method. Methods.

[43] Imbabi T.A., Ahmed-Farid O., Selim D.A., Sabeq I.I. (2021). Antioxidant and anti-apoptotic potential of whole-pomegranate extract promoted growth performance, physiological homeostasis, and meat quality of V-line rabbits under hot summer conditions. Anim. Feed. Sci. Technol..

[44] SAS Institute (1996). SAS/STAT Software: Changes and Enhancements for Release 6.12.

[45] Anwar F., Hussain A.I., Sherazi S.T.H., Bhanger M.I. (2009). Changes in Composition and Antioxidant and Antimicrobial Activities of Essential Oil of Fennel (*Foeniculum Vulgare* Mill.) Fruit at Different Stages of Maturity. J. Herbs Spices Med. Plants.

[46] Ertas O.N., Guler T., Çiftçi M., DalkIlIç B., Simsek U.G. (2005). The effect of an essential oil mix derived from oregano, clove and anise on broiler performance. Int. J. Poult. Sci..

[47] Benlemlih M., Aarab A., Bakkali M., Arakrak A., Laglaoui A. (2014). Effect of dietary fennel and thyme essential oil supplementation on zootechnical parameters and caecal microflora of growing rabbit. Rev. Microbiol. Ind. San Environ..

[48] Al-Kassie G.A.M., Abd-Al-Jaleel R.A., Mohseen A.M. (2011). The effect of a mixture of anise and rosemary on broiler performance. Agric. Biol. J. North Am..

[49] Schöne F., Vetter A., Hartung H., Bergmann H., Biertümpfel A., Richter G., Muller S., Breitschuh G. (2006). Effects of essential oils from fennel (Foeniculi aetheroleum) and caraway (Carvi aetheroleum) in pigs. J. Anim. Physiol. Anim. Nutr..

[50] Mohammed A.A., Abbas R.J. (2009). The Effect of Using Fennel Seeds (*Foeniculum Vulgare* L.) on Productive Performance of Broiler Chickens. Int. J. Poult. Sci..

[51] Singh P., Mishra N., Gupta E. (2020). Phytochemistry and ethanopharmacology of *Illicium verum* (Staranise). Interdisciplinary Approaches to Altering Neurodevelopmental Disorders.

[52] Elghalid O.A., Kholif A., El-Ashry G., Matloup O., Olafadehan O., El-Raffa A., El-Hady A.A. (2020). Oral supplementation of the diet of growing rabbits with a newly developed mixture of herbal plants and spices enriched with special extracts and essential oils affects their productive performance and immune status. Livest. Sci..

[53] Hernández F., Madrid J., García V., Orengo J., Megias M. (2004). Influence of two plant extracts on broilers performance, digestibility, and digestive organ size. Poult. Sci..

[54] Coelho-de-Souza A.N., Lahlou S., Barreto J.E., Yum M.E., Oliveira A.C., Oliveira H.D., Celedônio N.R., Feitosa R.G., Duarte G.P., Santos C.F. (2013). Essential oil of Croton zehntneri and its major constituent anethole display gastroprotective effect by increasing the surface mucous layer. Fundam. Clin. Pharmacol..

[55] De Oliveira Monteschio J., de Souza K.A., Vital A.C.P., Guerrero A., Valero M.V., Kempinski E.M.B.C., Barcelos V.C., Nascimento K.F., do Prado I.N. (2017). Clove and rosemary essential oils and encapsuled active principles (eugenol, thymol and vanillin blend) on meat quality of feedlot-finished heifers. Meat Sci..

[56] Ranucci D., Beghelli D., Trabalza-Marinucci M., Branciari R., Forte C., Olivieri O., Pazmay G.B., Cavallucci C., Acuti G. (2015). Dietary effects of a mix derived from oregano (*Origanum vulgare* L.) essential oil and sweet chestnut (*Castanea sativa* Mill.) wood extract on pig performance, oxidative status and pork quality traits. Meat Sci..

[57] Janz J., Morel P., Wilkinson B., Purchas R. (2007). Preliminary investigation of the effects of low-level dietary inclusion of fragrant essential oils and oleoresins on pig performance and pork quality. Meat Sci..

[58] Hunt M.C., King A., Barbut S., Clause J., Cornforth D., Hanson D., Lindahl G., Mancini R., Milkowski A., Mohan A. (2012). AMSA Meat Color Measurement Guidelines.

[59] Rodríguez-Calleja J.M., García-López M.-L., Santos J.A., Otero A. (2005). Development of the aerobic spoilage flora of chilled rabbit meat. Meat Sci..

[60] Parejo I., Jauregui O., Sánchez-Rabaneda F., Viladomat F., Bastida A.J., Codina C. (2004). Separation and Characterization of Phenolic Compounds in Fennel (*Foeniculum Vulgare*) Using Liquid Chromatography−Negative Electrospray Ionization Tandem Mass Spectrometry. J. Agric. Food Chem..

[61] Hyldgaard M., Mygind T., Meyer R.L. (2012). Essential Oils in Food Preservation: Mode of Action, Synergies, and Interactions with Food Matrix Components. Front. Microbiol..

[62] Imbabi T., Hassan A., Ahmed-Farid O., El-Garhy O., Sabeq I., Moustafa M., Mohammadein A., Hassan N., Osman A., Sitohy M. (2021). Supplementing rabbit diets with butylated hydroxyanisole affects oxidative stress, growth performance, and meat quality. Animal.

[63] Valko M., Leibfritz D., Moncol J., Cronin M.T.D., Mazur M., Telser J. (2007). Free radicals and antioxidants in normal physiological functions and human disease. Int. J. Biochem. Cell Biol..

[64] Mohamad R.H., El-Bastawesy A.M., Abdel-Monem M.G., Noor A.M., Al-Mehdar H.A.R., Sharawy S.M., El-Merzabani M.M. (2011). Antioxidant and anticarcinogenic effects of methanolic extract and volatile oil of fennel seeds (*Foeniculum Vulgare*). J. Med. Food.

[65] Linseisen J., Wolfram G. (1993). Odd-Numbered Medium-Chain Triglycerides (Trinonanoin) in Total Parenteral Nutrition: Effects on Parameters of Fat Metabolism in Rabbits. J. Parenter. Enter. Nutr..

[66] Cunha T.J., Cheeke P.R. (2012). Rabbit Feeding and Nutrition.

[67] Lee K.-W. (2002). Essential Oils in Broiler Nutrition. Ph.D. Thesis.

[68] Case G.L., He L., Mo H., Elson C.E. (1995). Induction of geranyl pyrophosphate pyrophosphatase activity by cholesterol-suppressive isoprenoids. Lipids.

[69] Sedláková J., Kocourková B., Lojková L., Kubáň V. (2003). The essential oil content in caraway species (*Carum carvi* L.). Hortic. Sci..

[70] Nazih H., Krempf M., Huvelin J.M., Mercier S., Bard J.M. (2001). Butyrate stimulates ApoA-IV-containing lipoprotein secretion in differentiated Caco-2 cells: Role in cholesterol efflux. J. Cell. Biochem..

[71] Podrez E. (2010). Anti-oxidant properties of high-density lipoprotein and atherosclerosis. Clin. Exp. Pharmacol. Physiol..

[72] Shahidullah A., Bhuiyan M., Hossain I., Islam R., Riaz M. (2016). Effects of gentamicin on growth performance and hemato-biochemical parameters in mice. Int. J. Nat. Soc. Sci..

[73] Hong J.-C., Steiner T., Aufy A., Lien T.-F. (2012). Effects of supplemental essential oil on growth performance, lipid metabolites and immunity, intestinal characteristics, microbiota and carcass traits in broilers. Livest. Sci..

[74] Wetterling T., Veltrup C., Driessen M., John U. (1999). Drinking pattern and alcohol-related medical disorders. Alcohol Alcohol..

[75] Onyesom I., Onyesom H.C., Opajobi A.O., Esume C.O. (2007). Effect of the permissive sociocultural consumption of alcohol on selected biochemical markers of liver function in the serum of some Nigerian drinkers. Adiktologie.

[76] Abdel-Hamid M., Osman A., El-Hadary A., Romeih E., Sitohy M., Li L. (2020). Hepatoprotective action of papain-hydrolyzed buffalo milk protein on carbon tetrachloride oxidative stressed albino rats. J. Dairy Sci..

[77] Osman A., Abd-Elaziz S., Salama A., Eita A.A., Sitohy M. (2019). Health protective actions of phycocyanin obtained from an Egyptian isolate of *Spirulina platensis* on albino rats. EurAsian J. Biosci..

[78] Kumar A. (2012). A review on hepatoprotective herbal drugs. Int. J. Res. Pharm. Chem..

[79] Nazir T., Shakir L., Rahman Z.-U., Najam K., Choudhary A., Saeed N., Rasheed H.-U., Nazir A., Aslam S., Khanum A.B. (2020). Hepatoprotective Activity of *Foeniculum Vulgare* Against Paracetamol Induced Hepatotoxicity in Rabbit. J. Appl. Pharm..

[80] Bovera F., Moniello G., de Riu N., Di Meo C., Pinna W., Nizza A. (2007). Effect of diet on the metabolic profile of ostriches (*Struthio camelus* var. domesticus). Trop. Anim. Health Prod..

[81] Moniello G., Bovera F., Solinas I., Piccolo G., Pinna W., Nizza A. (2005). Effect of age and blood collection site on the metabolic profile of ostriches (Short communication). South Afr. J. Anim. Sci..

[82] Miguel M.G. (2010). Antioxidant and Anti-Inflammatory Activities of Essential Oils: A Short Review. Molecules.

[83] Zhang S., Chen X., Devshilt I., Yun Q., Huang C., An L., Dorjbat S., He X. (2018). Fennel main constituent, trans-anethole treatment against LPS-induced acute lung injury by regulation of Th17/Treg function. Mol. Med. Rep..

[84] Liu Z., Ding Y., Ye N., Wild C., Chen H., Zhou J. (2016). Direct Activation of Bax Protein for Cancer Therapy. Med. Res. Rev..

[85] Naseri M.H., Mahdavi M., Davoodi J., Tackallou S.H., Goudarzvand M., Neishabouri S.H. (2015). Up regulation of Bax and down regulation of Bcl2 during 3-NC mediated apoptosis in human cancer cells. Cancer Cell Int..

[86] Yu Z.-Q., Jia Y., Chen G. (2014). Possible involvement of cathepsin B/D and caspase-3 in deferoxamine-related neuroprotection of early brain injury after subarachnoid haemorrhage in rats. Neuropathol. Appl. Neurobiol..

[87] Okuno S.-I., Shimizu S., Ito T., Nomura M., Hamada E., Tsujimoto Y., Matsuda H. (1998). Bcl-2 Prevents Caspase-independent Cell Death. J. Biol. Chem..

[88] Erşahin M., Özsavcı D., Şener A., Ozakpınar O.B., Toklu H.Z., Akakin D., Şener G., Yeğen B.Ç. (2013). Obestatin alleviates subarachnoid haemorrhage-induced oxidative injury in rats via its anti-apoptotic and antioxidant effects. Brain Inj..

[89] Tso M.K., Lass E., Ai J., Macdonald R.L. (2015). Valproic Acid Treatment after Experimental Subarachnoid Hemorrhage.

[90] Topkoru B.C., Altay O., Duris K., Krafft P.R., Yan J., Zhang J.H. (2013). Nasal Administration of Recombinant Osteopontin Attenuates Early Brain Injury after Subarachnoid Hemorrhage. Stroke.

[91] Sikiru A.B., Arangasamy A., Alemede I.C., Guvvala P.R., Egena S.S.A., Ippala J.R., Bhatta R. (2019). *Chlorella vulgaris* supplementation effects on performances, oxidative stress and antioxidant genes expression in liver and ovaries of New Zealand White rabbits. Heliyon.

