# Supplementary material for: Impact of Fennel Essential Oil as an Antibiotic Alternative in Rabbit Diet on Antioxidant Enzymes Levels, Growth Performance, and Meat Quality
Source: Antioxidants (Basel). 2021 Nov 10;10(11):1797. doi: 10.3390/antiox10111797 (PMC8614930; doi:10.3390/antiox10111797)
Supplement: Supplementary file 1 [file antioxidants-10-01797-s001.zip › antioxidants-1424469-supplementary.pdf]

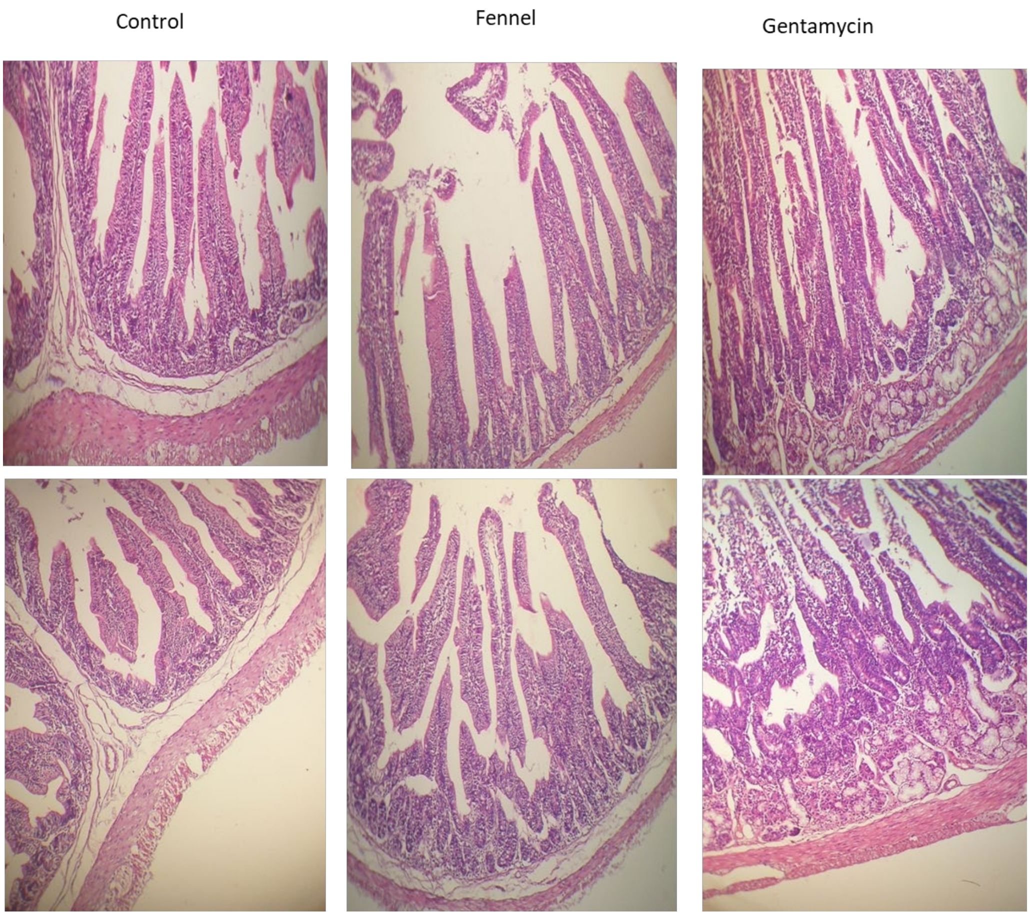

Figure S1. Villus morphology and morphometry characteristics of the small intestine in the rabbits receiving fennel oil and Gentamycin
